# Dr. James V. Kendall

**Published:** 1901-09

**Authors:** 


Dr. James V. Kendall, of Baldwinsville, N. Y., a graduate of Geneva Medical College, 1844, died August 5, 1901, aged 83 years.

